# The Natural History of Specific Diseases

**Published:** 1890-04-05

**Authors:** 


### THE NATURAL HISTORY OF SPECIFIC DISEASES.

Dr. Edward F. Willoughby writes: In giving to the world my speculations on "The Natural History of Specific Diseases," I intended to challenge criticism, and I should feel indebted to any one who would point out any fallacies in my reasoning, or suggest more satisfactory hypotheses. But I do expect my critics to take the trouble to ascertain my views before indulging in ridicule, and certainly not to misrepresent me.

Your reviewer entirely ignores the most important features of the work, viz., the attempt, the first ever made, at a rational and scientific classification of these diseases, involving the ideas of the extracorporeal perpetuation of the microbes of some, and the autochthonous origin of others, and the classification of prophylactic inoculations, also original; as well as my advocacy of Salmon's doctrine of immunity. These, with the application of evolution as solution of the vexed question of the first origin of specific diseases, constitute the essentials of the essay; all besides is but expansion, I might almost say padding.

But what I really complain of is that he has not merely missed but misrepresented my opinions; he makes me say that "*specific* diseases always result from the transmission of specific microbes from one individual to another," though I give a subgenus of miasmata, *not communicable*, co-ordinate with the contagia, and the subordinate division of extracorporeal contagia, may arise *de novo*, *i.e.*, the first of a series may be derived from extraneous sources, others following by infection from it.

Had he read the context with the care that a reviewer is bound in fairness to bestow on any book, he would have seen that by *ex nihilo*, I mean from no *material* virus. Diphtheria, for example, I hold to be occasionally produced *de novo*, *i.e.*, where no case has existed, but not, like catarrhal laryngitis, from exposure to cold, which certainly is *not a thing*, *i.e.*, is *nihil*. So, too, with enteric fever. I do not see anything "*amusing*" in this, and I invite the reviewer to suggest a better explanation of the dispute between Jenner and Murchison, whom I think I have reconciled by mine.

